# Cost-effectiveness of rilertinib versus osimertinib in second-line treatment in EGFR T790M resistance mutation advanced non-small cell lung cancer in China

**DOI:** 10.3389/fphar.2025.1628024

**Published:** 2025-10-09

**Authors:** Xiangcheng Li, Yun Lu, Yuqiong Lu, Mengyuan Zhou, Xiaoxi Xie, Yang Zhou, Jiaxin Qiu, Zhanjing Dai, Feng Chang

**Affiliations:** School of International Pharmaceutical Business, China Pharmaceutical University, Nanjing, China

**Keywords:** rilertinib, osimertinib, cost-effectiveness, non-small cell lung cancer, epidermal growth factor receptor, China

## Abstract

**Background:**

Rilertinib, a third-generation epidermal growth factor receptor (EGFR) tyrosine kinase inhibitor (TKI), has demonstrated a favorable efficacy and safety profile in adult patients with EGFR T790M + advanced NSCLC. This study examined the cost-effectiveness of rilertinib compared with osimertinib in the second-line treatment for EGFR T790M mutation-positive advanced NSCLC in the Chinese healthcare setting.

**Methods:**

A Markov model was developed to project economic and health outcomes. An unanchored matching-adjusted indirect comparison (MAIC) was used to compare the efficacy of rilertinib with osimertinib. Cost and utility values were obtained from Chinese health system data, public databases, and the published literature. Model robustness was assessed through deterministic and probabilistic sensitivity analyses (PSA).

**Results:**

The incremental life-years (LYs) and quality-adjusted life years (QALYs) for the rilertinib group versus the osimertinib group were 0.34 and 0.30, respectively. The total cost for the rilertinib group was $3,774.60 higher than that for the osimertinib group. The incremental cost-effectiveness ratio (ICER) for rilertinib group compared with osimertinib group was $12,786.08, which is lower than one time the GDP *per capita* ($13,444.68). Based on the willingness-to-pay (WTP) thresholds in China, rilertinib represented a cost-effective option. Relative efficacy and drug costs parameters were the key drivers of the model outcomes. PSA showed rilertinib’s cost-effective probabilities were 51.6% at one-time GDP *per capita* and 90.2% at three-times GDP *per capita* ($40,334.05) WTP threshold.

**Conclusion:**

From a Chinese healthcare system perspective, second-line treatment of EGFR T790M resistance mutation advanced NSCLC with rilertinib may have cost-effectiveness compared with osimertinib.

## 1 Introduction

Lung cancer is the predominant malignant tumor globally in terms of both incidence and mortality (Bray et al., 2024), posing a great threat to patient health. According to the latest statistics released by the National Cancer Center of China, the age-standardized incidence of lung cancer by the world standard population was 40.78/10^5^ and the age-standardized mortality by the world standard population was 26.66/10^5^ in 2022, both ranking first (Han et al., 2024). Non-small cell lung cancer (NSCLC) constitutes approximately 80%–85% of all lung cancer cases (Planchard et al., 2018; Gou and Wu, 2014; Bareschino et al., 2011), with a global 5-year overall survival rate of merely 16% (Li et al., 2021). Meanwhile, the economic burden on lung cancer patients is severe. In 2017, the aggregate economic burden attributable to lung cancer in China was estimated at 25.07 billion USD, representing 0.121% of China’s gross domestic product (GDP) (Liu et al., 2021). Projections suggest that this burden will increase to 53.40 billion USD by 2030 (Liu et al., 2021). Notably, drug costs constitute a primary component of this economic burden. In 2018, the average annual direct medical cost for lung cancer patients in China ranged from 7,766.15 to 10,874.14 USD, with the average cost per hospitalization spanning from 1,205.34 to 9,208.15 USD (Song et al., 2019). Drug costs accounted for the largest proportion (35.9% and 68.4% (Song et al., 2019)) of average cost per hospitalization.

Epidermal growth factor receptor (EGFR) mutations are the most prevalent driver mutations in Chinese patients with advanced NSCLC, accounting for 50.2% (Shi et al., 2015). Multiple Chinese guidelines recommend the use of EGFR tyrosine kinase inhibitors (TKIs) for the treatment of such patients (Huang et al., 2020; Zhi et al., 2025; Oncology Society of Chinese Medical Association, 2025). Among them, the Chinese Society of Clinical Oncology (CSCO) Guidelines for NSCLC (2025), the most authoritative NSCLC guideline in China, recommends EGFR-TKIs as the first-line therapy for patients with stage IV EGFR-mutant NSCLC (Huang et al., 2020). However, resistance against first- and second-generation EGFR TKIs inevitably emerges, typically resulting in a median progression-free survival (PFS) of only 9–14 months (Cheng et al., 2016). The most common mechanism of resistance is the development of the T790M secondary mutation, found in about 50% of patients treated with first- and second-generation TKIs (Soria et al., 2018). To address this challenge, third-generation EGFR TKIs (such as osimertinib and rilertinib) have been developed to effectively inhibit EGFR T790M–positive tumors and overcome resistance to earlier-generation EGFR-TKIs, extending the patients’ PFS and overall survival (OS) (Wu et al., 2018). Therefore, they are strongly recommended by the CSCO Guidelines for NSCLC (2025) as the second-line treatment. In addition to osimertinib and rilertinib, several other third-generation EGFR-TKIs, including aumolertinib, furmonertinib, befotertinib, and rezivertinib are also recommended in Chinese clinical guidelines for the second-line treatment. This increasingly competitive environment underscores the importance of evaluating the relative value and cost-effectiveness of each new option.

Rilertinib is a Class 1.1 innovative drug and third-generation EGFR-TKI independently developed by a Chinese pharmaceutical company. It has been approved in China by the National Medical Products Administration (NMPA) and was included in the 2024 National Reimbursement Drug List (NRDL). In a recent multicenter, single-arm, open-label Phase II clinical trial (SHC013-II-01, NCT03823807) (Xiong et al., 2022) conducted in China, rilertinib demonstrated promising efficacy in 227 patients with EGFR T790M mutation–positive NSCLC. According to assessments by the independent review committee (IRC), rilertinib achieved an objective response rate (ORR) of 60.8% (95% confidence interval [CI] 54.1%–67.2%), a median PFS of 12.2 months (95% CI 9.7-13.8), and a median OS of 25.9 months (95% CI 25.1-not available [NA]). Besides, the common treatment-emergent adverse events (TEAEs) of grade ≥3 (occurring in ≥1% of patients) included increased serum creatinine phosphokinase (4.5%), diarrhea (2.1%), upper respiratory tract infection (1.0%), and prolonged electrocardiogram QT interval (1.0%).

Cost-effectiveness is one of the most critical drug attributes in addition to the efficacy and safety. Although the clinical efficacy and safety of rilertinib has been established (Xiong et al., 2022), its cost-effectiveness as a scond-line therapy remains uncertain. Consequently, evaluating the cost-effectiveness is critical for understanding the true impact of rilertinib and choosing the optimal option among various alternatives with limited resources. Therefore, in accordance with the CSCO Guidelines for NSCLC (2025) (Guidelines of Chinese Society of Clinical Oncology CSCO, 2025) and the Chinese Guidelines for Pharmacoeconomic Evaluations (Liu et al., 2020), this study selects osimertinib, which has the same indication and a high recommendation level, as the comparator to conduct an economic evaluation of rilertinib. This evaluation aims to inform value-based clinical decision making, reduce patients’ economic burden, and provide a scientific basis for future work such as the renewal of the National Reimbursement Drug List (NRDL).

## 2 Materials and methods

### 2.1 Model structure

In accordance with the Consolidated Health Economic Evaluation Reporting Standards 2022 (CHEERS 2022) (Husereau et al., 2022), this study conducted a cost-utility analysis based on quality-adjusted life year (QALY), which are widely accepted as the most commonly used outcome measure in cost-utility analysis, to evaluate the cost-effectiveness of rilertinib versus osimertinib from the Chinese healthcare system’s perspective.

**FIGURE 1:**
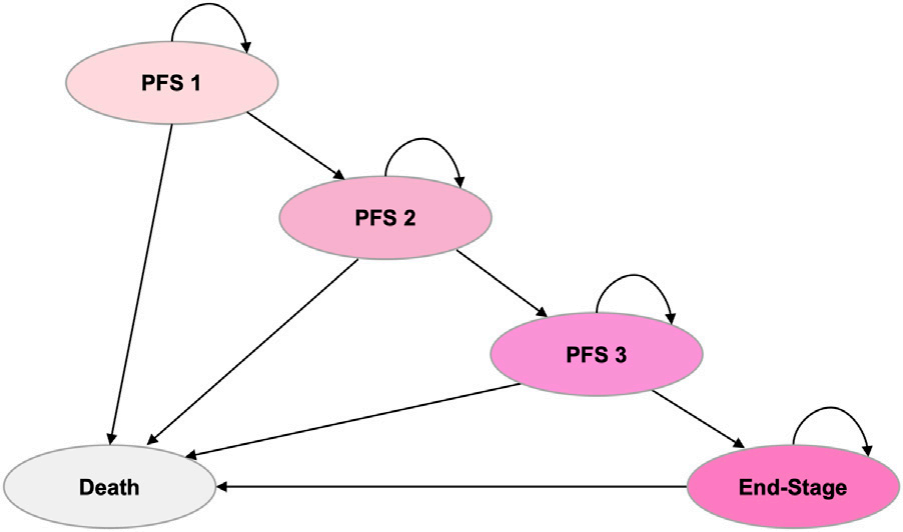
Markov model structure. The model includes five health states: PFS 1, PFS 2, PFS 3, end-stage, and death. Arrows indicate possible transitions between states. PFS, progression-free survival.

Considering the clinical characteristics of EGFR + NSCLC, such as its long disease course, high propensity for metastasis, and clear treatment sequencing, we have developed a five-state Markov model that considers second-line and sequential treatment regimens using Microsoft Excel for Mac 16.71 (Microsoft Corporation, Redmond, WA). The states included PFS 1, PFS 2, PFS 3, end-stage, and death (Figure 1).

All patients entered the model in the PFS one state, where those in the intervention group received rilertinib and those in the control group received osimertinib. The model simulated the disease progression process and was validated through expert consultation to ensure its accuracy in reflecting real-world clinical practice. Both rilertinib and osimertinib are third-generation EGFR-TKIs that share a pyrimidine-based core structure. The structural modification of the indole ring in rilertinib may contribute to a more favorable safety profile. However, at present, there is no clinical evidence suggests that this structural difference leads to a distinct acquired resistance profile compared with osimertinib. Therefore, similar resistance mechanisms were assumed for both treatment groups. Based on clinical guidelines and expert advice, identical subsequent treatment regimens were applied in the PFS two and PFS three states: both groups received bevacizumab combined with pemetrexed and carboplatin in the PFS two state, and anlotinib combined with docetaxel in the PFS three state.

The number of patients in each PFS state (PFS 1–3) during each cycle was determined by the respective PFS curves corresponding to their treatment regimens. Patients who experienced disease progression in any PFS state transitioned to the subsequent state. Mortality in the death state incorporated the natural mortality rate of the Chinese population plus treatment-related adverse event mortality. The end-stage state included patients progressing in PFS 3. At any given cycle, patients occupied only one state.

The base-case analysis adopted a 15-year time horizon, representing a lifetime perspective for these patients, as the mortality rates for both groups were approximately 98.6% (rilertinib) and 99.6% (osimertinib). The model simulation used a starting age of 61 years, consistent with the mean age of patients in the rilertinib trial, with surviving patients projected to approach the average life expectancy of the Chinese population in 2021 (78.2 years) (Xinwen, 2022).

Costs and QALYs were discounted at 5% per annum in accordance with the China Guidelines for Pharmacoeconomics Evaluation 2020 (Liu et al., 2020). Costs incurred in previous years were adjusted using the consumer price index of 2005–2024 and were expressed in 2024 US dollars (1 USD = 7.12 CNY).

In this analysis, a willingness-to-pay (WTP) threshold of $40,334.05 per QALY—equivalent to three times the *per capita* GDP of China—was used to determine cost-effectiveness. The threshold was based on the China Guidelines for Pharmacoeconomics Evaluation 2020 (Liu et al., 2020) and supported by published studies (Thokala et al., 2018; Cameron et al., 2018; Tzanetakos and Gourzoulidis, 2023), which define treatments as cost-effective if the ICER is less than three times the GDP *per capita* ($40,334.05). Furthermore, a treatment is considered highly cost-effective if the ICER is less than one times the GDP *per capita* ($13,444.68). The estimated *per capita* GDP of China was $13,444.68 (CNY 95,749), derived from the 2024 Statistical Communiqué on National Economic and Social Development of the People’s Republic of China (The Statistical Communiqué, 2025).

Given the treatment cycles of rilertinib and subsequent therapies, and PFS data reported in rilertinib clinical trials at 3-month intervals, the cycle length for this model was set as 3 months. Disease progression was simulated through discrete cycles by dividing time into successive intervals; this discretization process could introduce minor inaccuracies in outcome and cost calculations. To mitigate such errors, a half-cycle correction was applied to refine the estimation.

### 2.2 Data sources, inputs, and modeling

#### 2.2.1 Efficacy data

PFS data for rilertinib were estimated from the individual patient data (IPD) provided by the Sanhome pharmaceutical company, based the single-arm SHC013-II-01 trial (Xiong et al., 2022) in PFS one state. This study enrolled eligible adult patients with locally advanced or metastatic NSCLC who had experienced disease progression during or after previous EGFR TKIs treatment and were confirmed to have EGFR T790M mutation-positive through testing. Efficacy data for osimertinib were obtained from the AURA3 trial (Mok et al., 2017; Papadimitrakopoulou et al., 2020), the latest phase 3 trial evaluating osimertinib as second-line therapy, using the same eligibility criteria. In PFS two state, efficacy data were derived from the PointBreak trial (Patel et al., 2013), in which all patients received bevacizumab combined with pemetrexed and carboplatin. In PFS three state, efficacy data were derived from the ALTER 0303 trial (Han et al., 2018), in which all patients received anlotinib combined with docetaxel.

Due to the absence of head-to-head studies comparing rilertinib with osimertinib, indirect comparisons were required in this study. Given the availability of IPD of rilertinib but only aggregate data (AgD) of osimertinib, the unanchored matching-adjusted indirect comparison (MAIC) method (Phillippo et al., 2016) was used, as there was no common comparator in SHC013-II-01 and AURA3. For the selection of matching variables, we referred to the published literature employing MAIC, Cox analysis results, clinical opinions, and the availability of baseline characteristics. Ultimately, six variables were selected for MAIC: age, sex, smoking status, central nervous system (CNS) metastasis at baseline, Exon 19 deletion mutation, and L858R mutation. The Cox analysis results and the rationale for selecting these matching variables were presented in Supplementary Tables S1, S2, respectively. The selection of matching variables was based on the intersection of key prognostic factors reported in both the SHC013-II-01 trial IPD and the published AURA3 trial AgD (Mok et al., 2017; Papadimitrakopoulou et al., 2020). Consequently, variables such as race could not be included, as this information was unavailable in the rilertinib IPD. Similarly, ECOG performance status was not included as it was not reported in the AURA3 publication. However, the inclusion criteria for both trials restricted enrollment to patients with “locally advanced or metastatic NSCLC”, indicating substantial comparability in disease stage between the two cohorts. The final baseline characteristics before and after matching are shown in Table 1. The effective sample size (ESS) was 74.38; this metric is calculated to assess the validity of the matching process, with a larger value indicating a better balance. The detailed principles and calculation formula are described in Supplementary Methods 1.

**TABLE 1:** Baseline characteristics before and after MAIC adjustment.

Matching variables	Rilertinib (SHC013-II-01)		Osimertinib (AURA3)
	Before	After	
N/ESS	N = 227	ESS = 74.38	
Age [Mean (SD)]	61 (9.57)	62 (15.0)	62 (15.0)
Male (%)	42.73%	38.35%	38.35%
No history of smoking (%)	74.44%	67.74%	67.74%
CNS metastasis at baseline (%)	35.24%	33.33%	33.33%
Exon 19 deletion mutation (%)	67.40%	67.40%	68.46%
L858R mutation (%)	34.36%	34.36%	29.75%

ESS, effective sample size; SD, standard deviation; CNS, central nervous system; N, number of patients.

The PFS curve for rilertinib was constructed based on the available IPD. The PFS curve of osimertinib was constructed based on reconstructed IPD data from the clinical trials. To reconstruct the data, this study used GetData Graph Digitizer and the algorithm by Guyot et al. (2012). Subsequently, the hazard ratio (HR) of PFS between the two interventions was calculated using the matching weights. After MAIC adjustment, the PFS-HR of rilertinib versus osimertinib was estimated at 0.763 (standard deviation [SD]: 0.155, 95% CI: 0.527 - 1.104).

To extrapolate the long-term efficacy of each treatment, this study used five parametric survival models recommended by NICE (Latimer, 2011) guidance documents (Exponential, Weibull, Gompertz, Log-logistic, and Lognormal) for curve fitting. Model selection was based on clinical plausibility, visual fit, and statistical goodness-of-fit [Akaike information criterion (AIC) and Bayesian information criterion (BIC)], where lower values indicate a better model fit. For PFS one state, the log-logistic distribution was selected to model the PFS curve for rilertinib, with the osimertinib’s PFS curve was derived by applying the HR adjustment. For PFS two state, the log-normal distribution was selected for the PFS curve for bevacizumab combined with pemetrexed and carboplatin. For PFS three state, the log-logistic distribution was selected for the PFS curve for anlotinib combined with docetaxel (AIC and BIC values for each PFS were shown in Supplementary Table S3; parametric survival curve fittings were presented in Supplementary Figures S1–S3). Additionally, the model integrated age-specific mortality reported in the 2020 China Population Census Yearbook (China Population Census Yearbook, 2020), along with mortality from treatment-related adverse events, to simulate survival outcomes more accurately.

#### 2.2.2 Utility inputs

QALYs were determined through health state utility values (HSUVs). These HSU values were individually computed for patients in the PFS and PD states. An assumption was made that utility values remained consistent across different treatment groups. HSU values of PFS and PD were sourced from a study of health state utilities in NSCLC conducted by Nafees et al. (2017), since the original trials of both interventions did not measure patients’ quality of life. We extracted the China-specific utility values, and the values of PFS 1, PFS two and PFS three states were 0.804 while the value of end-stage state was 0.321 in the base–case analysis. Furthermore, disutilities associated with treatment-related adverse events (AEs) with a severity of grade ≥ 3 and an incidence of ≥1% were integrated into the model. Because these AEs were expected to meaningfully reduce the quality of life, grade 1/2 AEs were generally self-limited (Lu et al., 2023). The AEs incidence of rilertinib was provided by Sanhome. Given osimertinib’s extensive clinical use and well-documented safety profile, which are comprehensively reported in its package insert, AEs and their incidence for the osimertinib arm were derived from the drug’s package insert in the base-case analysis.

These disutility values were derived from existing published research and were limited to the initial treatment cycles. Detailed information on utility values used in this study is shown in Table 2. For AEs with unavailable disutility data, a disutility of 0 was assumed.

**TABLE 2:** Key utility parameters and their variations.

Parameters	Value	Lower limit	Upper limit	Distribution	Data source
Utility values					
PFS	0.804	0.643	0.965	Beta	Nafees et al. (2017)
PD	0.321	0.257	0.385	Beta	Nafees et al. (2017)
Disutility of AEs					
Prolonged QT	−0.06	−0.07	−0.06	Beta	Sivignon et al. (2020)
Diarrhea	−0.07	−0.08	−0.06	Beta	Nafees et al. (2017)
URI	0	0	0	Beta	Assumed
Neutropenia	−0.20	−0.22	−0.18	Beta	Nafees et al. (2017)
Anemia	−0.07	−0.08	−0.07	Beta	Wan et al. (2019)
Thrombocytopenia	−0.11	−0.12	−0.10	Beta	Tolley et al. (2013)
Leukopenia	−0.20	−0.22	−0.18	Beta	Tang et al. (2023)
Increased CK	0	0	0	Beta	Assumed
Vomiting	−0.12	−0.13	−0.11	Beta	Nafees et al. (2017)
Decreased Appetite	−0.05	−0.05	−0.04	Beta	Holleman et al. (2020)
Increased ALT	0	0	0	Beta	Assumed
Increased AST	0	0	0	Beta	Assumed
Fatigue	−0.07	−0.08	−0.06	Beta	Nafees et al. (2017)
Nausea	−0.12	−0.13	−0.11	Beta	Nafees et al. (2017)
Dyspnea	−0.27	−0.29	−0.24	Beta	Holleman et al. (2020)
Rash	−0.10	−0.11	−0.09	Beta	Nafees et al. (2017)
Headache	0	0	0	Beta	Assumed
Asthenia	−0.07	−0.08	−0.06	Beta	Nafees et al. (2017)
ILD	−0.17	−0.19	−0.15	Beta	Simons et al. (2021)
Lymphopenia	−0.11	−0.12	−0.10	Beta	Palmer and Leeuwenkamp (2020), Smith-Palmer et al. (2021)

PFS, progression-free survival; PD, progression disease; AEs, adverse events; URI, upper respiratory infection; CK, creatine kinase; ALT, alanine aminotransferase; AST, aspartic transaminase; ILD, interstitial lung disease.

#### 2.2.3 Resource use and costs

This study calculated costs from a China’s healthcare system perspective, including only direct medical costs (Liu et al., 2020) (Table 3). Most costs were derived from the published literature, and on this basis, opinions of clinical experts were considered. For drugs with multiple specifications, prices were standardized to per-milligram cost to facilitate the calculation.

1 Drug costs

**TABLE 3:** Key cost parameters and their variations.

Parameters	Value	Lower limit	Upper limit	Distribution	Data source
Drug costs, per unit, $					
Rilertinib (100 mg)	13.93	12.54	15.32	Gamma	NHSA (National Healthcare Security Administration), Pharnexcloud database (Pharnexcloud)
Osimertinib (80 mg)	23.25	20.93	25.58	Gamma	MENET database (MENET)
Bevacizumab (1 mg)	1.77	1.59	1.94	Gamma	MENET database (MENET)
Pemetrexed (1 mg)	0.61	0.55	0.67	Gamma	MENET database (MENET)
Folic Acid (1 mg)	0.16	0.14	0.17	Gamma	MENET database (MENET)
Vitamin B12 (1 mg)	3.85	3.47	4.24	Gamma	MENET database (MENET)
Dexamethasone (1 mg)	0.78	0.70	0.86	Gamma	MENET database (MENET)
Carboplatin (1 mg)	0.17	0.15	0.18	Gamma	MENET database (MENET)
Anlotinib (12 mg)	43.53	39.18	47.89	Gamma	MENET database (MENET)
Docetaxel (1 mg)	2.62	2.36	2.88	Gamma	MENET database (MENET)
Monitoring costs, per unit, $					
Outpatient	2.64	2.3	2.90	Gamma	Service Price Catalogue (Service Price Catalogue, 2025)
Blood routine examination	4.61	4.15	5.08	Gamma	Service Price Catalogue (Service Price Catalogue, 2025)
Blood biochemistry examination	8.87	7.99	9.76	Gamma	Service Price Catalogue (Service Price Catalogue, 2025)
Urine routine examination	0.39	0.35	0.43	Gamma	Service Price Catalogue (Service Price Catalogue, 2025)
Electrocardiogram	4.12	3.71	4.53	Gamma	Service Price Catalogue (Service Price Catalogue, 2025)
Magnetic resonance imaging	75.63	68.06	83.19	Gamma	Service Price Catalogue (Service Price Catalogue, 2025)
Chest CT	54.51	49.06	59.96	Gamma	Service Price Catalogue (Service Price Catalogue, 2025)
Serum creatine kinase test	1.93	1.74	2.13	Gamma	Service Price Catalogue (Service Price Catalogue, 2025)
D-Dimer test	7.76	6.99	8.54	Gamma	Service Price Catalogue (Service Price Catalogue, 2025)
Hospitalization and caring costs, per day, $					
Best supportive care	115.15	103.64	126.67	Gamma	Li et al. (2015)
Palliative care	26.36	23.72	28.99	Gamma	Li et al. (2018)
Hospitalization and care	163.23	146.91	179.56	Gamma	Chen et al. (2014)
AEs cost, $					
Prolonged QT	8.27	7.44	9.09	Gamma	Liu and He (2022)
Diarrhea	6.08	5.47	6.69	Gamma	Guan et al. (2019)
URI	0	0	0	Gamma	Assumed
Neutropenia	104.18	93.76	114.59	Gamma	Li et al. (2012)
Anemia	41.63	37.47	45.80	Gamma	Yu et al. (2021)
Thrombocytopenia	143.82	129.44	158.20	Gamma	Zhang et al. (2015)
Leukopenia	88.46	79.62	97.31	Gamma	Wang et al. (2015)
Increased CK	55.02	49.52	60.52	Gamma	Guo et al. (2019)
Vomiting	64.78	58.31	71.26	Gamma	Zhang et al. (2015)
Decreased Appetite	19.84	17.85	21.82	Gamma	Liu and He (2022)
Increased ALT	42.55	38.30	46.81	Gamma	Guo et al. (2019)
Increased AST	22.72	20.45	25.00	Gamma	Feng et al. (2018)
Fatigue	0	0	0	Gamma	Assumed
Nausea	0	0	0	Gamma	Assumed
Dyspnea	0	0	0	Gamma	Assumed
Rash	11.02	9.92	12.12	Gamma	Zhu et al. (2018)
Headache	5.43	4.88	5.97	Gamma	Li et al. (2005)
Asthenia	0	0	0	Gamma	Assumed
ILD	1,625.16	1462.65	1787.68	Gamma	Simons et al. (2021)
Lymphopenia	70.87	63.78	77.96	Gamma	Xu et al. (2019)
Hypertension	97.94	88.15	107.74	Gamma	Zheng et al. (2018)
Proteinuria	121.97	109.78	134.17	Gamma	Zheng et al. (2018)
Administration cost, per unit, $					
Intravenous administration	1.14	1.02	1.25	Gamma	Service Price Catalogue (Service Price Catalogue, 2025)
Intramuscular administration	0.47	0.42	0.52	Gamma	Service Price Catalogue (Service Price Catalogue, 2025)

CT, computed tomography; AEs, adverse events; URI, upper respiratory infection; CK, creatine kinase; ALT, alanine aminotransferase; AST, aspartic transaminase; ILD, interstitial lung disease.

In PFS one state, it was assumed that subjects would not incur additional drug administration costs, as both rilertinib and osimertinib are oral drugs. The latest price of rilertinib was 13.93 USD/100 mg (National Healthcare Security Administration, 2025; Pharnexcloud database, 2025), while the price of osimertinib was 23.25 USD/80 mg (MENET, 2025). In the PFS two state, the drug costs encompassed not only the expenses of bevacizumab injection, pemetrexed disodium injection, and carboplatin, but also covered prophylactic drugs (folic acid, vitamin B12, dexamethasone), along with the costs associated with administering non-oral drugs. In PFS three state, the drug costs involved anlotinib, docetaxel injection, prophylactic dexamethasone, and the charges for non-oral drug administration. Detailed information on drug costs in this study is shown in Table 3.

2 Monitoring, hospitalization and caring costs

This study referenced the CSCO Guidelines for NSCLC (2025) (Guidelines of Chinese Society of Clinical Oncology CSCO, 2025), the SHC013-II-01 clinical trial data provided by the company (Sanhome), the package inserts of rilertinib and osimertinib, and expert surveys to define the items of monitoring in PFS 1. Monitoring items for each treatment were determined based on their respective package inserts. Unit costs for these items were derived from Service Price Catalogue (Table 3). According to clinical expert consensus, patients progressing to PFS two or PFS three states require hospitalization, with examination costs included in “hospitalization and care costs”; best supportive care costs were also incorporated into these two states. Costs in the end-stage state primarily comprised “palliative care costs”. Costs associated with best supportive care, palliative care, and hospitalization and care cost were derived from published literature (Table 3). The frequencies of detailed monitoring, hospitalization and caring are shown in Supplementary Tables S4 and S5.

3 AEs costs

This study included adverse event costs based on the clinical trials and package insert, and only considered the management caused by AEs with a severity of grade ≥3 and an incidence of ≥1% as they were expected to result in significant healthcare utilization (Lu et al., 2023). AE management costs were limited to the initial treatment cycles and calculated once. Unit costs for AEs were derived from published sources or assumed, and the unit cost, and source of AEs are shown in Table 3. The incidence rates of AEs for each drug are shown in Supplementary Table S6.

### 2.3 Sensitivity analysis

A comprehensive uncertainty assessment was conducted through a series of sensitivity analyses to identify key drivers of model outcomes. For deterministic sensitivity analysis (DSA), each parameter was individually varied within ±10% of the baseline value or within the 95% confidence interval, with the results presented in tornado diagrams. In PSA, multivariate parameter sampling was executed through 1,000 iterations of Monte Carlo simulation. Uncertainty in the HRs of PFS was estimated with normal distributions, health state utility followed beta distributions, and costs were assigned gamma distributions. The results of the PSA were presented through cost-effectiveness acceptability curves (CEACs) and probabilistic scatter plots. This study also conducted scenario analysis, and the scenarios were shown in Table 5.

## 3 Results

### 3.1 Base-case analysis

The results of the base–case analysis are presented in Table 4. For rilertinib, the mean costs and QALYs were $141,390.08 and 2.36 respectively, while for osimertinib, the mean costs and QALYs were $137,615.49 and 2.07 respectively. The ICER for rilertinib versus osimertinib is $12,786.08/QALY, which falls below the threshold of 1 times China’s *per capita* GDP, indicating that rilertinib is highly cost-effective treatment option.

**TABLE 4:** The results of base–case analysis.

Interventions	Rilertinib	Osimertinib	Increment
Cost			
Drug and administration costs	$110,191.31	$107,519.75	$2,671.55
Monitoring, hospitalization and caring costs	$31,141.36	$30,015.44	$1,125.92
AEs costs	$57.41	$80.29	-$22.88
Total costs	$141,390.08	$137,615.49	$3,774.60
LYs	3.25	2.91	0.34
QALYs	2.36	2.07	0.30
ICER			$12,786.08

AEs, adverse events; QALY, quality-adjusted life-year; LY, life year; ICER, incremental cost-effectiveness ratio.

### 3.2 Sensitivity analysis

**FIGURE 2:**
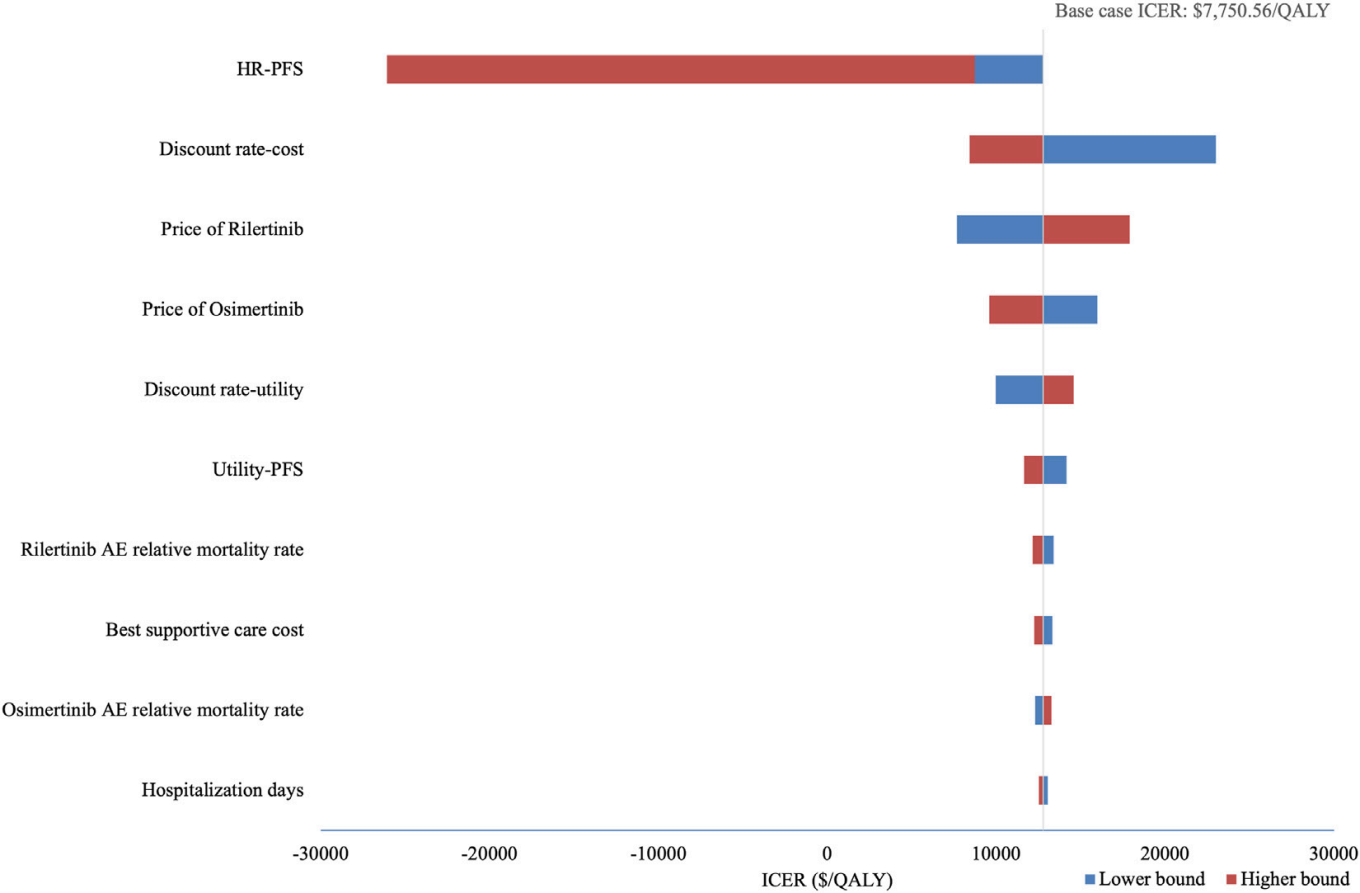
Tornado diagram of deterministic sensitivity analysis. This diagram illustrates the impact of key parameters on the ICER. The vertical line represents the base-case ICER, and horizontal bars show the range of ICERs when varying each parameter. HR, hazard ratio; PFS, progression-free survival; AE, adverse event; ICER, incremental cost-effectiveness ratio.

Ten of the most influential parameters in the DSA are illustrated in Figure 2. Among all parameters, the HR for PFS of rilertinib versus osimertinib has the largest impact on the ICER: when the HR took the lower limit (0.527), the ICER was $8,738.45; when the HR took the upper limit (1.104), the ICER became -$26,080.72, indicating the cost-effectiveness result would reverse. Therefore, the naive indirect comparison results were incorporated into the scenario analysis for comprehensive evaluation. After the PFS HR and discount rate for costs, the ICER is most sensitive to the prices of rilertinib and osimertinib.

**FIGURE 3:**
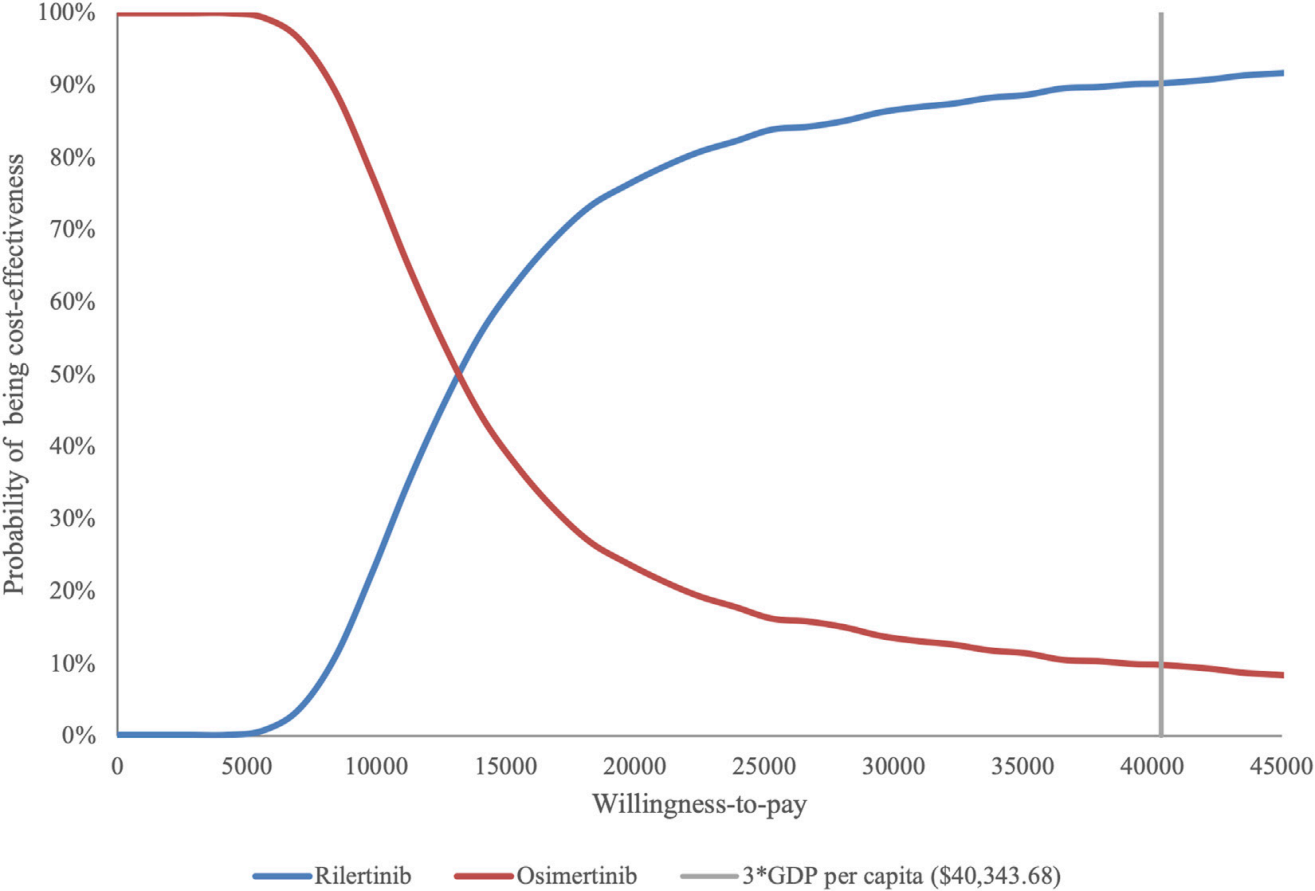
Cost-effectiveness acceptability curve. The curve shows the probability of rilertinib being cost-effective at various WTP thresholds. WTP, willingness-to-pay; GDP, gross domestic product.

**FIGURE 4:**
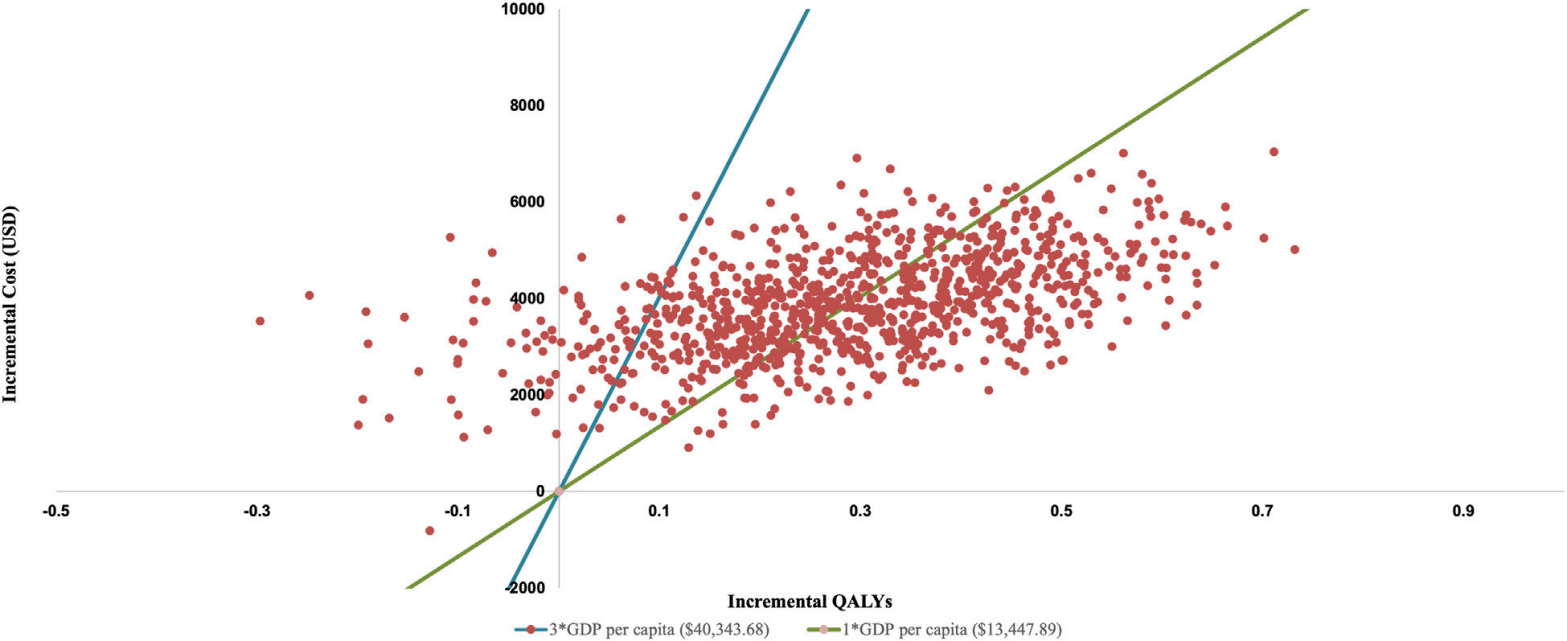
Probabilistic scatter plot of the ICER between rilertinib and osimertinib. Each point represents the results of a single PSA iteration. QALY, quality adjusted life year; GDP, gross domestic product.

PSA show an average QALY gain of 0.291 and an incremental cost of $3,886.63, resulting in a probabilistic ICER of $13,361.88/QALY which is consistent with the base-case analysis result. The CEAC is shown in Figure 3, which indicates that when the WTP threshold exceeds $13,146.07, rilertinib has a greater than 50% probability of being the cost-effective option. The scatter plot in Figure 4 visualizes the outcome of all PSA simulations. Most simulations fell within the first quadrant, associating rilertinib with increased costs and QALYs. Notably, 90.20% of these points lie below the three-times GDP *per capita* threshold (blue line), and 51.60% below the one-time GDP threshold (green line). This distribution provides supportive evidence for the cost-effectiveness of rilertinib.

The results of the scenario analysis are detailed in Table 5. In all scenarios, the ICERs are below 3 times China’s *per capita* GDP ($40,343.68), with the ICER in Scenario two falling below 1 times the *per capita* GDP ($13,447.89). Cost-effectiveness trends are robust across all variables in both the sensitivity and scenario analyses.

**TABLE 5:** The results of scenario analysis.

Scenario description	Cost/QALYs		ICER
	Rilertinib	Osimertinib	
Base-case analysis	$141,390.08/2.36	$137,615.49/2.07	$12,786.08
Naïve indirect comparison	$141,390.08/2.36	$138,066.15/2.15	$16,066.30
Include AEs based on AURA3	$141,390.08/2.36	$137,595.29/2.07	$12,886.61
Lognormal distribution for Rilertinib PFS	$141,689.70/2.35	$137,757.32/2.07	$13,839.88
20-year time horizon	$142,191.42/2.38	$137,904.16/2.07	$13,772.96

AEs, adverse events; QALY, quality-adjusted life-year; ICER, incremental cost-effectiveness ratio; PFS, progression-free survival.

## 4 Discussion

In recent years, China has witnessed rapid development of innovative anticancer drugs, which have significantly improved patient outcomes. According to the Center for Drug Evaluation (CDE) of China (NMPA, 2024), anti-tumor drugs represent the largest category among all approved drugs. However, their high cost may limit patient accessibility (Hao et al., 2021) pose a threat to the long-term sustainability of the healthcare system (Migliorino et al., 2017). Economic evidence has been institutionalized as a critical component in China’s annual national drug reimbursement negotiations, allowing for dynamic updates to the National Reimbursement Drug List (NRDL) and thereby improving the accessibility and affordability of innovative therapies (Gao and Liu, 2017).

In this study, the mean total costs and QALYs for rilertinib were $141,390.08 and 2.36 respectively, compared with $137,615.49 and 2.07 for osimertinib. The ICER for rilertinib versus osimertinib was $12,786.08/QALY, falling below the threshold of one time China’s *per capita* GDP, indicating that rilertinib is highly cost-effective treatment option. In addition to its favorable economic profile, rilertinib demonstrated robust clinical efficacy.

To the best of our knowledge, this study represents the first cost-effectiveness analysis comparing rilertinib versus osimertinib, both the first-in-class and third-generation EGFR-TKI, for treating locally advanced or metastatic NSCLC in adult patients with confirmed EGFR T790M mutation after progression on prior EGFR TKIs therapy. Given that rilertinib’s approval was based on single-arm trial data, an unanchored MAIC was employed. This approach, recommended by NICE for indirect treatment comparisons, was used to balance the baseline characteristics between two trial populations and reduce potential bias in survival data caused by uneven covariate distribution. Previous studies have shown that NSCLC patients harboring canonical EGFR mutations (Exon 19 deletion or L858R) derive greater benefit from third-generation EGFR-TKIs (Gomez-Randulfe et al., 2025; Gristina et al., 2020; Borgeaud et al., 2024) with mutation subtypes significantly affecting cost-effectiveness estimates (Tian et al., 2024). Therefore, in this study, the proportions of canonical mutations were aligned across treatment groups via the MAIC approach, further mitigating potential efficacy bias.

DSA revealed that the hazard ratio (HR) for PFS had the greatest impact on the model outcomes. Accordingly, a scenario analysis was conducted, including a naïve indirect comparison in Scenario 1, which confirmed that the ICER remained below three times the GDP *per capita*, with no reversal of cost-effectiveness. PSA further supported the robustness of the base-case findings, showing that rilertinib had a 90.20% probability of being cost-effective at the WTP threshold, reinforcing the robustness of the base-case results. Across all scenario analysis, no ICER exceeded the 3 times GDP *per capita*, reinforcing the reliability of the conclusions.

To date, no published study has evaluated the cost-effectiveness of rilertinib as a second-line treatment for EGFR T790M-positive advanced NSCLC. Although previous research has established osimertinib’s cost-effectiveness in the second-line setting (Guan et al., 2019; Shi et al., 2022), even after several rounds of price reduction in China, this analysis demonstrates that rilertinib maintains cost-effectiveness. These findings the economic value of rilertinib for Chinese patients with EGFR T790M-positive advanced NSCLC.

This study has several advantages. First, given that rilertinib’s second-line indication has already been included in the NRDL, this analysis provides timely pharmacoeconomic evidence supporting its continued inclusion, offering guidance for clinical decision-making and reimbursement policy. Second, a five-state sequential Markov model was developed based on real-world clinical pathways, informed by expert consultation, and covering multiple lines of therapy through to death. This approach provides a comprehensive economic framework for evaluating long-term treatment outcomes. Third, treatment efficacy was compared using a rigorous MAIC methodology, ensuring balanced comparison in the absence of head-to-head trials. Fourth, extensive sensitivity analyses confirmed the robustness of the model.

Nonetheless, this study has several limitations. First, although MAIC was used to adjust for observed baseline differences between rilertinib and osimertinib trial populations, residual confounding from unobserved prognostic factors may remain. For instance, variables such as ECOG performance status and race could not be matched, as they were not reported in the AURA3 or were unavailable in the IPD of rilertinib. Future head-to-head randomized controlled trials are needed to validate these results. Second, utility data were derived from literature, using methods consistent with prior studies (Wu et al., 2018; Guan et al., 2019; Wu et al., 2019), which may introduce uncertainty. However, sensitivity analysis demonstrated that the ICER remained stable and below the threshold. Third, the model adopted protocol-specified dosing rather than real-world practices patterns, potentially affecting resource use estimates. Despite this, a series of sensitivity analysis confirmed the robustness of the model across a broad range of parameter values. Fourth, subgroup analyses based on potential differences in acquired resistance mechanisms or patient histology were not performed due to limited evidence. Nevertheless, the unified post-progression pathway in our model reflects the standard of care for the overall T790M-positive population, an approach supported by major clinical guidelines as well as expert consultation. These assumptions are considered reasonable and are unlikely to affect the study’s main conclusions.

Based on the findings and limitations, future research should focus on the following aspects. First, as the clinical use of rilertinib increases, it will be essential to incorporate emerging real-world data to update and validate the simulation, for providing more comprehensive evidence. Second, real-world evidence collection should specifically investigate potential differences in resistance mechanisms between third-generation EGFR-TKIs and their impacts on clinical outcomes to further refine treatment pathways and economic evaluations. Third, evidence on key patient subgroups should be accumulated to enable future subgroup analyses and provide more nuanced cost-effectiveness evidence.

## 5 Conclusion

This study utilized clinical trial data (Xiong et al., 2022; Mok et al., 2017; Papadimitrakopoulou et al., 2020) to perform a robust indirect comparison of efficacy and incorporated local resource utilization and unit cost data to model the cost-effectiveness of rilertinib. The results suggest that, compared with osimertinib, rilertinib may be associated with longer PFS and yielded greater LYs and QALYs. For adult patients with locally advanced or metastatic EGFR T790M mutation-positive NSCLC who progressed after prior EGFR TKIs, rilertinib could represent a cost-effective treatment option based on the available evidence.

## Data Availability

The original contributions presented in the study are included in the article/Supplementary Material, further inquiries can be directed to the corresponding authors.

## Glossary

EGFR: Epidermal growth factor receptor
AE: Adverse event
AgD: Aggregate data
AIC: Akaike information criterion
BIC: Bayesian information criterion
CDE: Center for Drug Evaluation
CEAE: Cost-effectiveness acceptability curves
CI: Confidence interval
CNS: Central nervous system
DSA: Deterministic sensitivity analysis
ESS: Effective sample size
GDP: Gross domestic product
HR: Hazard ratio
HSUV: health state utility value
ICER: Incremental cost-effectiveness ratio
IPD: Individual patient data
IRC: Independent review committee
MAIC: Matching-adjusted indirect comparison
NA: Not available
NHSA: National Healthcare Security Administration
NMPA: National Medical Products Administration
NRDL: National Reimbursement Drug List
NSCLC: Non-small cell lung cancer
ORR: Objective response rate
OS: Overall survival
PFS: Progression-free survival
PSA: Probabilistic sensitivity analyses
QALY: Quality-adjusted life years
SD: Standardized deviation
TEAE: Treatment-emergent adverse events
TKI: Tyrosine kinase inhibitor
TTO: Time trade-off
WTP: Willingness-to-pay
