# Supplementary material for: Cost-effectiveness of rilertinib versus osimertinib in second-line treatment in EGFR T790M resistance mutation advanced non-small cell lung cancer in China
Source: Front Pharmacol. 2025 Oct 9;16:1628024. doi: 10.3389/fphar.2025.1628024 (PMC12545111; doi:10.3389/fphar.2025.1628024)
Supplement: Supplementary file 1 [file Supplementaryfile1.docx]

Appendix

**Supplementary Figure 1** KM and parametric survival curve fits for PFS of Rilertinib

**Supplementary Figure 2** KM and parametric survival curve fits for PFS of PemCBev

**Supplementary Figure 3** KM and parametric survival curve fits for PFS of Anlotinib + Docetaxel

## **Figure 1 KM and parametric survival curve fits for PFS of Rilertinib**

The stepped line represents the observed KM survival data. The smooth lines represent different parametric models used for extrapolation. This visualization allows for an assessment of model goodness-of-fit.

## **Figure 2 KM and parametric survival curve fits for PFS of PemCBev**

The stepped line represents the observed KM survival data for bevacizumab combined with pemetrexed and carboplatin. The smooth lines represent different parametric models used for extrapolation.

## **Figure 3 KM and parametric survival curve fits for PFS of Anlotinib + Docetaxel**

The stepped line represents the observed KM survival data for anlotinib combined with docetaxel. The smooth lines represent different parametric models used for extrapolation.
