# Supplementary material for: Cost-effectiveness of rilertinib versus osimertinib in second-line treatment in EGFR T790M resistance mutation advanced non-small cell lung cancer in China
Source: Front Pharmacol. 2025 Oct 9;16:1628024. doi: 10.3389/fphar.2025.1628024 (PMC12545111; doi:10.3389/fphar.2025.1628024)
Supplement: Supplementary file 2 [file Supplementaryfile3.docx]

Appendix

**Supplementary Table S1** The result of Cox analysis.

**Supplementary Table S2** Matching variable selection of the unanchored MAIC.

**Supplementary Table S3** Parametric functions fitting for PFS of Rilertinib.

**Supplementary Table S4** Monitoring frequency of Rilertinib and Osimertinib.

**Supplementary Table S5** Duration and frequency of hospitalization in PFS 2 and PFS 3 state.

**Supplementary Table S6** Incidence of AEs.

## **Table S1 The result of Cox analysis**

| **Variables** | **Single-factor** | | **Multi-factor** | |
| --- | --- | --- | --- | --- |
|  | **HR** | **p** | **HR** | **p** |
| Age | 0.98 | 0.066 | 0.98 | **0.045*** |
| Sex | 1.50 | **0.044*** | 1.30 | 0.250 |
| Smoking status | 1.40 | **0.099.** | 1.30 | 0.370 |
| Histopathologic type | 0.48 | 0.210 | 0.39 | 0.110 |
| CNS metastasis at baseline | 1.50 | **0.039*** | 1.50 | **0.046*** |
| Exon 19 deletion mutation | 0.73 | 0.12 | 1.50 | 0.360 |
| L858R mutation | 1.50 | **0.032*** | 2.20 | **0.069.** |

“***”, p ＜ 0.001;“**”, p ＜ 0.01;“*”, p ＜ 0.05;“.”, p ＜0.1; CNS, central nervous system.

## **Table S2 Matching variable selection of the unanchored MAIC**

| **Variables** | **In/Excluded** | **Reasons** |
| --- | --- | --- |
| Age | included | - Published researches^[1][2][3][5][6][7][8]^ took age as a matching variable in the unanchored MAIC - COX analysis stated that age as a prognostic variable significantly affected the efficacy |
| Sex | included | - Published researches^[1][2][3][4][8]^ took sex as a matching variable in the unanchored MAIC - COX analysis stated that sex as a prognostic variable significantly affected the efficacy |
| Smoking status | included | - Published researches^[1][3][4][8]^ took smoking status as a matching variable in the unanchored MAIC - COX analysis stated that smoking status as a prognostic variable significantly affected the efficacy |
| Histopathologic type | excluded | - The histopathologic type classification criteria utilized in the Osimertinib trial (AURA3) differ methodologically from those adopted in the Rilertinib trial - COX analysis did not state that histopathologic type as a prognostic variable significantly affected the efficacy |
| CNS metastasis at baseline | included | - Published researches^[1][2]^ took CNS metastasis at baseline as a matching variable in the unanchored MAIC, and Reckamp et al.^[1]^ considered CNS metastasis at baseline as a prognostic variable - COX analysis stated that CNS metastasis at baseline as a prognostic variable significantly affected the efficacy |
| Exon 19 deletion mutation | included | - Published research shows that mutant targets in lung cancer have a great impact on the therapeutic efficacy^[9]^ |
| L858R mutation | included | - COX analysis stated that L858R mutation as a prognostic variable significantly affected the efficacy - Published research shows that mutant targets in lung cancer have a great impact on the therapeutic efficacy^[9]^ |

## **Table S3 Parametric functions fitting for PFS of Rilertinib**

| **Model** | **Rilertinib-PFS**  **(PFS 1 - Rilertinib)** | | **PemCBev-PFS**  **(PFS 2 – both groups)** | | **Anlotinib+Doc-PFS**  **(PFS 3 – both groups)** | |
| --- | --- | --- | --- | --- | --- | --- |
|  | **AIC** | **BIC** | **AIC** | **BIC** | **AIC** | **BIC** |
| Exponential | 487.1402 | 490.5428 | 107.1954 | 108.8842 | 767.2531 | 770.9434 |
| Weibull | 477.3942 | 484.1995 | 104.3955 | 107.7733 | 719.3355 | 726.7162 |
| Gompertz | 485.1257 | 491.931 | 107.8179 | 111.1956 | 745.3637 | 752.7445 |
| Log-normal | 470.1875 | 476.9928 | 100.0117 | 103.3894 | 719.1562 | 726.5369 |
| Log-logistic | 472.1210 | 478.9263 | 100.6229 | 104.0007 | 718.7987 | 726.1794 |

PFS, progression-free survival; PemCBev, Pemetrexed plus Carboplatin and bevacizumab; Anlotinib+Doc, Anlotinib plus Docetaxel.

## **Table S4 Monitoring frequency of Rilertinib and** **Osimertinib**

|  | **Rilertinib** | **Osimertinib** |
| --- | --- | --- |
| Outpatient | Once every 2 weeks | Once every 2 weeks |
| Blood routine examination | Once every 2 weeks | Once every 2 weeks |
| Blood biochemistry examination | Once every 2 weeks | Once every 2 weeks |
| Urine routine examination | Once every 2 weeks | Once every 2 weeks |
| Electrocardiogram | Once every 2 weeks | Once every 2 weeks |
| Magnetic resonance imaging | Once every 2 weeks | Once every 2 weeks |
| Chest CT | Once every 2 weeks | Once every 2 weeks |
| Serum creatine kinase test | Once every 2 weeks | N/A |
| D-Dimer test | Once every 2 weeks | N/A |

CT, computed tomography; N/A: Not Applicable.

## **Table S5 Duration and frequency of hospitalization in PFS 2 and PFS 3 state**

|  | **PFS 2** | **PFS 3** |
| --- | --- | --- |
| Duration of hospitalization | 10.4 days^[10]^ | 10.4 days^[10]^ |
| Frequency of hospitalization | Once every 1 month^[11]^ | Once every 1 month^[11]^ |

PFS, progression-free survival.

## **Table S6 Incidence of AEs**

| **AE** | **Incidence** |
| --- | --- |
| Rilertinib (from SHC013-II-01^[12]^) | |
| Prolonged QT | 1.00% |
| Diarrhea | 2.10% |
| URI | 1.00% |
| Neutropenia | 0.70% |
| Anemia | 0.70% |
| Thrombocytopenia | 0.70% |
| Leukopenia | 0.70% |
| Increased CK | 4.50% |
| Vomiting | 0.30% |
| Osimertinib (from package insert) | |
| Diarrhea | 1.40% |
| Neutropenia | 3.20% |
| Leukopenia | 1.20% |
| Thrombocytopenia | 1.20% |
| Lymphopenia | 6.10% |
| ILD | 1.10% |
| Osimertinib (from AURA3^[13,14]^) | |
| Diarrhea | 1.00% |
| Decreased Appetite | 2.00% |
| Increased ALT | 2.00% |
| Increased AST | 1.00% |
| Neutropenia | 3.00% |
| Anemia | 1.00% |
| Leukopenia | 1.00% |
| Thrombocytopenia | 1.00% |
| Vomiting | 1.00% |
| Fatigue | 1.00% |
| Nausea | 1.00% |
| Dyspnea | 1.00% |
| Rash | 1.00% |
| Headache | 1.00% |
| Asthenia | 1.00% |
| Pemetrexed plus carboplatin and bevacizumab (from Zhang et al.^[15]^) | |
| Neutropenia | 14.00% |
| Hypertension | 11.70% |
| Proteinuria | 4.70% |
| Anemia | 4.70% |
| Leukopenia | 5.40% |
| Anlotinib plus Docetaxel (from Ma et al.^[16]^) | |
| Neutropenia | 12.84% |

AEs, adverse events; URI, upper respiratory infection; CK, creatine kinase; ALT, alanine aminotransferase; AST, aspartic transaminase; ILD, Interstitial lung disease.

# References

1. Reckamp KL, Lin HM, Cranmer H, Wu Y, Zhang P, Kay S, et al. Overall survival indirect treatment comparison between brigatinib and alectinib for the treatment of front-line anaplastic lymphoma kinase-positive non-small cell lung cancer using data from ALEX and final results from ALTA-1L. Curr Med Res Opin. (2022)38(9):1587-1593. doi:10.1080/03007995.2022.2100653
2. Li J, Knoll S, Bocharova I, Tang W, Signorovitch J. Comparative efficacy of first-line ceritinib and crizotinib in advanced or metastatic anaplastic lymphoma kinase-positive non-small cell lung cancer: an adjusted indirect comparison with external controls. Curr Med Res Opin. (2019)35(1):105-111. doi: 10.1080/03007995.2018.1541443
3. Tan DS, Araújo A, Zhang J, Signorovitch J, Zhou ZY, Cai X, et al. Comparative Efficacy of Ceritinib and Crizotinib as Initial ALK-Targeted Therapies in Previously Treated Advanced NSCLC: An Adjusted Comparison with External Controls. J Thorac Oncol. (2016)11(9):1550-1557. doi: 10.1016/j.jtho.2016.05.029
4. Smith S, Albuquerque de Almeida F, Inês M, Iadeluca L, Cooper M. Matching-Adjusted Indirect Comparisons of Lorlatinib Versus Chemotherapy for Patients With Second-Line or Later Anaplastic Lymphoma Kinase-Positive Non-Small Cell Lung Cancer. Value Health. (2023)26(1):64-70. doi: 10.1016/j.jval.2022.07.002
5. Halmos B, Burke T, Kalyvas C, Vandormael K, Frederickson A, Piperdi B. Pembrolizumab+chemotherapy versus atezolizumab+chemotherapy+/-bevacizumab for the first-line treatment of non-squamous NSCLC: A matching-adjusted indirect comparison. Lung Cancer. 2021;155:175-182. doi:10.1016/j.lungcan.2021.03.020.
6. Paik PK, Pfeiffer BM, Vioix H, Garcia A, Postma MJ. Matching-Adjusted Indirect Comparison (MAIC) of Tepotinib with Other MET Inhibitors for the Treatment of Advanced NSCLC with MET Exon 14 Skipping Mutations. Adv Ther. 2022;39(7):3159-3179. doi:10.1007/s12325-022-02163-9.
7. Halmos B, Burke T, Kalyvas C, et al. Indirect comparison of pembrolizumab monotherapy versus nivolumab + ipilimumab in first-line metastatic lung cancer. Immunotherapy. 2022;14(5):295-307. doi:10.2217/imt-2021-0273.
8. Halmos B, Burke T, Kalyvas C, et al. A Matching-Adjusted Indirect Comparison of Pembrolizumab + Chemotherapy vs. Nivolumab + Ipilimumab as First-Line Therapies in Patients with PD-L1 TPS ≥1% Metastatic NSCLC. Cancers (Basel). 2020;12(12):3648. Published 2020 Dec 4. doi:10.3390/cancers12123648
9. Majeed U, Manochakian R, Zhao Y, Lou Y. Targeted therapy in advanced non-small cell lung cancer: current advances and future trends. J Hematol Oncol. 2021;14(1):108. Published 2021 Jul 8. doi:10.1186/s13045-021-01121-2
10. National Health Commission of the People’s Republic of China . Available at: http://www.nhc.gov.cn/mohwsbwstjxxzx/tjtjnj/202501/b8d57baa95834269b5b3562bfec801a7.shtml (Accessed April 10, 2025)
11. Guidelines of Chinese Society of Clinical Oncology (CSCO) . Non-small Cell Lung Cancer. Beijing: People’s Medical Publishing House; (2024).
12. Xiong A, Ren S, Liu H, et al. Efficacy and Safety of SH-1028 in Patients With EGFR T790M-Positive NSCLC: A Multicenter, Single-Arm, Open-Label, Phase 2 Trial. J Thorac Oncol. 2022;17(10):1216-1226. doi:10.1016/j.jtho.2022.06.013
13. Mok TS, Wu Y-L, Ahn M-J, et al. Osimertinib or Platinum-Pemetrexed in EGFR T790M-Positive Lung Cancer. N Engl J Med. 2017;376(7):629-640. doi:10.1056/NEJMoa1612674
14. Papadimitrakopoulou VA, Mok TS, Han JY, et al. Osimertinib versus platinum-pemetrexed for patients with EGFR T790M advanced NSCLC and progression on a prior EGFR-tyrosine kinase inhibitor: AURA3 overall survival analysis. Ann Oncol. 2020;31(11):1536-1544. doi:10.1016/j.annonc.2020.08.2100

Zhang Q, Ding Y, Dai B, et al. Cost-Effectiveness Analysis of Pemetrexed Combined with Bevacizumab in Maintenance Therapy of Advanced Non - Squamous Non-Small–Cell Lung Cancer. China Pharmaceuticals. 2022,31(10),106-111 doi:10.3969/j.issn.1006-4931.2022.10.026 **(in Chinese)**

Ma H, Ren Z, Shang F, et al. Efficacy and Safety of Anlotinib Combined with Docetaxel in Second-line Treatment of Advanced Non-small Cell Lung Cancer with Negative Driver Gene. The Practical Journal of Cancer. 2023,38(03),476-480+484 doi:10．3969 / j.issn.1001-5930.2023.03.032 **(in Chinese)**
