# Supplementary material for: Cost-effectiveness of rilertinib versus osimertinib in second-line treatment in EGFR T790M resistance mutation advanced non-small cell lung cancer in China
Source: Front Pharmacol. 2025 Oct 9;16:1628024. doi: 10.3389/fphar.2025.1628024 (PMC12545111; doi:10.3389/fphar.2025.1628024)
Supplement: Supplementary file 3 [file Supplementaryfile2.docx]

Appendix

**Supplementary Method 1** Unanchored MAIC and ESS Calculation.

# Supplementary Material

## **Methods S1 Unanchored MAIC and ESS Calculation.**

**Principles of weight calculation**

The unanchored matching-adjusted indirect comparison (MAIC) was employed because individual patient data (IPD) were available for the rilertinib trial (Trial A), while only aggregate data (AgD) were available for the osimertinib trial (Trial B), and no common comparator existed. The core principle of MAIC is to re-weight the IPD from Trial A to create a "pseudo-population" that matches the baseline characteristics of the population in Trial B^[1]^.

This is achieved by calculating a weight for each individual in the rilertinib IPD. The weight ($w_{iA}$) is the ratio of the probability of an individual being enrolled in Trial B versus Trial A, estimated using a logistic regression model:

$$\log\left( w_{iA} \right)=\alpha_{0}+\alpha_{1}^{T}X_{iA} (Formula 1)$$

Where $X_{iA}$ is the vector of matching variables (e.g., age, sex, smoking status) for the i-th individual in Trial A. Since only AgD is available for Trial B, the regression parameters are estimated using the method of moments to precisely balance the means of the covariates between the two groups.

**Rationale and Calculation of Effective Sample Size (ESS)**

The ESS is calculated to formally evaluate the validity of the matching process. It represents the size of the re-weighted patient cohort from Trial A. A larger ESS value indicates a better matching effect and a higher degree of overlap between the two original trial populations. Conversely, a smaller ESS suggests that the populations were very different, requiring substantial re-weighting, which increases the uncertainty (i.e., widens the confidence intervals) of the final efficacy estimate^[1]^.

The ESS for an unanchored MAIC is calculated using the following formula:

$$ESS=\frac{{(\sum_{i=1}^{N_{A}} w_{iA})}^{2}}{\sum_{i=1}^{N_{A}} {w_{iA}}^{2}} (Formula 2)$$

Where $N_{A}$ is the original sample size of Trial A, and $w_{iA}$ is the weight calculated for the i-th individual in Trial A.

# References

1. Jiayi X, Zhanjing D, Yuqiong L et al. Analysis of the methodological points and practice status of matching-adjusted indirect comparison application. Chinese Journal of Evidence-Based Medicine. 2024,24(3):322-330. doi:10.7507/1672-2531.202310035 **(in Chinese)**
